# OpenDose3D: A Free, Open-Source Clinical Dosimetry Software for Patient-Specific Dosimetry

**DOI:** 10.2967/jnumed.125.269539

**Published:** 2026-02

**Authors:** José-Alejandro Fragoso-Negrín, Alex Vergara-Gil, Assyifa Rahman Hakim, Deni Hardiansyah, Gerhard Glatting, Ludovic Ferrer, Nicolas Varmenot, Lore Santoro, Susana Veloza-Awad, Kevin Hébert, Emmanuel Deshayes, Manuel Bardiès

**Affiliations:** 1 Institut de Recherche en Cancérologie de Montpellier, Équipe Labellisée Ligue Contre le Cancer, INSERM U1194, Institut Régional du Cancer de Montpellier, Université de Montpellier, Montpellier, France;; 2 Nuclear Medicine Department, Institut Régional du Cancer de Montpellier, Montpellier, France;; 3 DOSIsoft, Cachan, France;; 4 BIOMEDIQA GROUPE, Villeneuve-d’Ascq, France;; 5 Medical Physics and Biophysics, Physics Department, Faculty of Mathematics and Natural Sciences, Universitas Indonesia, Depok, Indonesia;; 6 Medical Radiation Physics, Department of Nuclear Medicine, Ulm University, Ulm, Germany; and; 7 Medical Physics Department, ICO René Gauducheau, Nantes, France

**Keywords:** patient-specific dosimetry, clinical dosimetry workflows, open-source software, radionuclide therapy, research methods

## Abstract

Software tools for nuclear medicine dosimetry are becoming available. Yet, it is difficult to compare clinical dosimetry workflows implemented in academic or commercial software tools, and this limits the emergence of a standardized clinical dosimetry practice. This article introduces OpenDose3D (OD3D), an open-source software designed to provide image-based, patient-specific dosimetry in nuclear medicine. OD3D implements multiple clinical dosimetry workflows and exports intermediate results, enabling comparison between various dosimetry approaches. **Methods:** OD3D was developed as an extension of the 3D Slicer platform that already presents several components adapted to nuclear medicine imaging. Three additional modules were created: a calibration module for deriving sensitivity factors and recovery coefficients for partial-volume effect correction, a module for calculating absorbed dose rate images based on 3 algorithms, and a module for fitting and integrating time-dependent variables (activity or absorbed dose rates). The software was tested against phantom data from the MRTDosimetry project and image data from the 2021 SNMMI Dosimetry Challenge dataset. **Results:** The phantom-based validation process showed excellent agreement (deviations <3%) with the reference values for calibration and organ dosimetry. For the tumor compartment, the difference in absorbed doses was attributed to pharmacokinetic modeling. The OD3D results using data from the SNMMI Dosimetry Challenge (patient A) were consistently close to the reported values for the 3 proposed tasks: absolute *z* score was less than 1. **Conclusion:** The OpenDose collaboration provides free dosimetry tools and data to the community. The OD3D software is a research tool that can be used for clinical dosimetry, to educate professionals, and to benchmark available academic or commercial clinical dosimetry software tools.

## Visual Abstract

**Figure:**
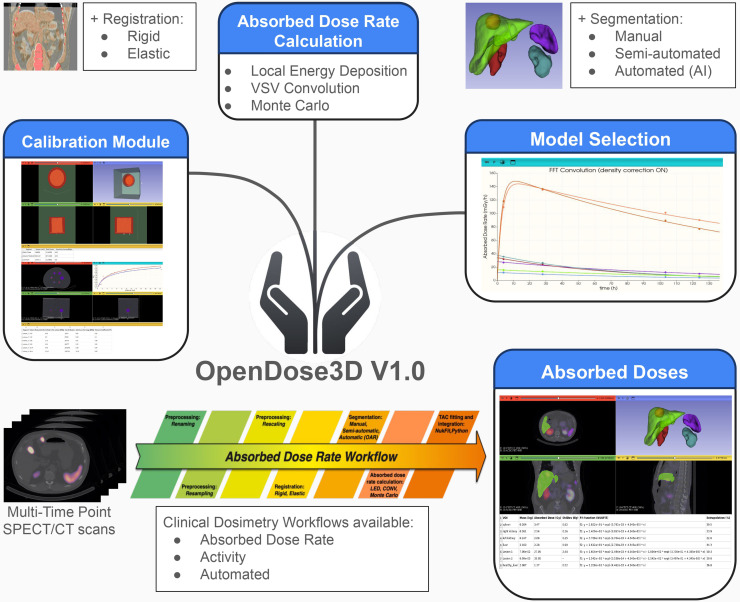

Clinical dosimetry is expanding in therapeutic nuclear medicine. For decades, clinical dosimetry software tools were confined to the academic world and developed for research purposes in specialized centers. More recently, commercial software tools, with Conformité Européenne marking or Food and Drug Administration clearance, have been proposed for routine clinical use.

Clinical dosimetry depends on the clinical objective. In diagnostic nuclear medicine, model-based dosimetry is implemented to document the irradiation delivered. Patient pharmacokinetic data are pooled, and precomputed *S* values obtained from anthropomorphic models are used to perform absorbed dose calculations (1–4).

For therapeutic applications, patient-specific dosimetry is recommended. The first step is to derive patient-specific pharmacokinetic data. Reference *S* values can be adjusted to the patient geometry for self-absorbed dose determination. However, fully patient-specific dosimetry is achieved by deriving both activity and absorbed doses from patient images (activity and density, obtained from SPECT/PET and CT images, respectively) (5). Currently, various academic or commercial software tools are available to the community for reference and patient-specific dosimetry. Their comparison and commissioning are not easy because they may implement different clinical dosimetry workflows (CDW) (6). Previous studies (7,8) compared some commercial software tools and proposed a “wish list” of missing features, some of which are implemented in the present project.

The OpenDose3D (OD3D) software belongs to the broader OpenDose collaboration (9), the aim of which is to provide free dosimetry resources for the nuclear medicine community. OD3D was developed as an open-source tool for retrospective dosimetry. OD3D allows performing fully patient-specific image-based dosimetry in a research context, based on reconstructed SPECT/CT or PET/CT images. Its open and modular structure allows benchmarking other software tools at various CDW steps. OD3D is based on the 3D Slicer Python application programming interface and hosted as a GitLab project (https://gitlab.com/opendose/opendose3d) to enable collaborative development. This article presents the first stable release (version 1.0).

## MATERIALS AND METHODS

OD3D is an extension of 3D Slicer (10), an open-source, general image display and processing software that includes a range of user-provided extensions to provide a comprehensive tool for medical image processing.

As many components required for performing dosimetry were already present in 3D Slicer, this tool was considered the basis for OD3D. Therefore, OD3D includes preexisting and specifically developed modules, assembled in a user-friendly graphical interface.

OD3D was developed as a suite of sequentially available modules. It uses reconstructed Digital Imaging and Communications in Medicine (DICOM) images from either SPECT/CT or PET/CT scans as input. The interface intuitively guides the user through each step of the workflow, and features become available when the previous steps are completed.

### Calibration Module

OD3D includes a standalone calibration module that generates the conversion factor needed to convert count-indexed images into activity images and a recovery coefficient (RC) curve which allows partial-volume effect (PVE) correction.

For camera sensitivity calibration (11), the module uses SPECT/CT images of a Jaszczak phantom uniformly filled with a measured activity. Input parameters include radionuclide, activity, acquisition duration, radioactive solution volume, and cylindric diameter; some of these are extracted from DICOM metadata, whereas others must be entered manually. Sensitivity is derived from SPECT-based automatic segmentation on total field of view, exact source volume, or extended source volume (12).

For RC calibration, the module uses National Electrical Manufacturers Association (NEMA) phantom images with 6 spheres of known volume and activity concentration. The sensitivity factor is required to calculate the activity within the spheres. Seed points are manually placed at sphere centers, followed by automatic segmentation on CT or SPECT. RCs are fitted using a 2-parameter logistic function (13) to correct PVE. The fitted RC curve can be used at the volume of interest (VOI) level, but this step is not automated within OD3D. Users are encouraged to apply PVE correction outside OD3D if required for their workflow.

Other phantoms can also be used for sensitivity factor and RC curve generation. Images and results of the calibration modules can be stored. RC can be used for PVE correction. Sensitivity information is automatically transferred to the OD3D main module.

### CDW

Two CDW approaches have been implemented in OD3D: activity (ACT) and absorbed dose rate (ADR). In both CDWs, time-indexed images are registered to a reference CT image. The VOIs are defined on the same reference and propagated according to the transformations defined during the registration process. Then, depending on the CDW, some steps are performed in a different order (Table 1).

**TABLE 1.** CDW Implementation in OD3D

CDW	Activity		Absorbed Dose Rate	
Mode	Manual	Automated	Manual	Automated
Registration	Rigid, elastic	Elastic*	Rigid, elastic	—
Segmentation	Manual, semiautomated, automated^†^	Automated*^,^^†^	Manual, semiautomated, automated^†^	Automated^†^
Absorbed dose rate	—	—	LED, CONV, MC	LED
Time integration	NUKFIT^‡^, Python	Default = NUKFIT	NUKFIT^‡^, Python	Default = NUKFIT
Absorbed dose	LED	LED	—	—

* By default, reference image is first time point.

† Using TotalSegmentator, only for CT-defined organs at risk.

‡ Fitting module selection is set in preference file.

#### ACT Workflow

Time–activity curves are calculated, fitted, and integrated to provide the time-integrated activity (TIA) in each VOI. Then, the mean absorbed dose is computed by multiplying the TIA (MBq·h) by the average electron per β-energy emitted per decay. The result is divided by the VOI reference mass (from the reference CT image).

#### ADR Workflow

Here, ADRs are computed at the voxel level, for each time point, for the whole field of view. The mean ADRs in each VOI are extracted, generating time–ADR curves that are then fitted and integrated to yield the mean absorbed dose in each VOI.

ADR maps are stored at each time point and can be exported for further use (e.g., isodoses, ADR volume histogram generation). Regardless of the workflow, curve fitting and integration are performed at the organ (VOI) level.

### Sequence of Operations

The 3D Slicer *Add Dicom Data* module is used first to upload phantom and patient data (i.e., reconstructed SPECT/CT or PET/CT images). Then, the OD3D module can be launched.

#### Initialization

**FIGURE 1.**
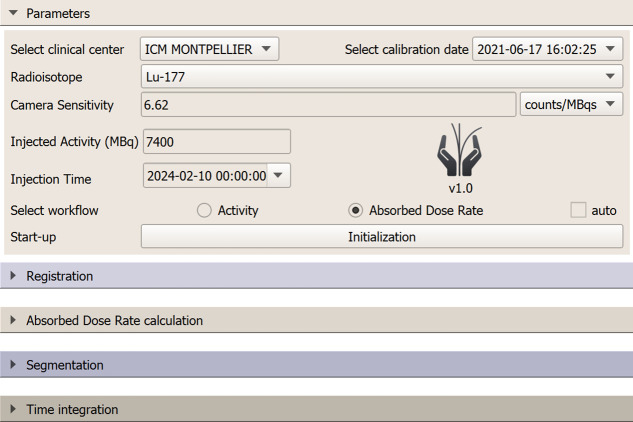
OpenDose3D version 1.0 Welcome page. Interface allows users to select clinical center settings, radioisotope, camera sensitivity, injected activity, and injection time. The following clinical dosimetry workflows are available for selection: Activity and Absorbed Dose Rate, with optional automated mode (auto). Expandable sections guide users through workflow steps (Registration, Absorbed Dose Rate calculation, Segmentation, and Time integration).

The initialization step requires uploading data in the Welcome page (Fig. 1). If the calibration module is not used, the sensitivity factor must be entered manually.

The radionuclides currently supported in OD3D are ^64^Cu, ^67^Cu, ^18^F, ^68^Ga, ^166^Ho, ^124^I, ^131^I, ^111^In, ^177^Lu, ^153^Sm, ^161^Tb, ^99m^Tc, and ^90^Y. The list can be extended if requested via the GitLab repository.

The injected activity and administration time are extracted from the DICOM header. If not present, a warning window asks users to enter this information.

The Welcome page also displays the software version to facilitate traceability. Users must choose between the ACT and ADR workflows and can select an automated, predefined sequence of operations (auto).

#### Preprocessing

Preprocessing is an automated sequence of operations that reorganizes data files and prepares the patient dataset for clinical dosimetry. Images are renamed and placed in specific folders per time point. CT images are resampled to SPECT spatial sampling; SPECT and CT images are transformed into activity and density-indexed images, respectively; see the Parameters section of the OD3D User’s Manual).

#### Registration and Segmentation

These steps are performed using preexisting 3D Slicer modules called in OD3D. A reference CT image is selected, and rigid or elastic registration is performed on the whole field of view using the 3D Slicer *Elastix* module (14).

The Segmentation module offers different possibilities:

- 3D Slicer can import preexisting segmentations as DICOM RT structures, when processing is made in several sessions or if externally segmented VOIs are available.
- Manual or semiautomated segmentation can be performed by calling *Segment Editor*. Segmentation is performed on a user-chosen reference image. This method is often used to create VOIs for lesions.
- Automated segmentation of organs at risk (OARs) is possible with TotalSegmentator (15). Segmentation is performed on the reference image. The structures to segment are defined in a preference file that can be adapted to various clinical situations. In addition, OD3D includes a tool for extracting the marrow region from vertebrae within the field of view, using Hounsfield unit thresholding and the segmentation provided by the TotalSegmentator module.

Segmentation is then propagated from the reference image to all time points using the transformation defined in the registration step.

Alternatively, VOI segmentation can be performed at each time point without registering the CT images. This approach may provide more accurate VOIs and positions that reflect the anatomy at each time point. This means accepting VOI changes at each time point.

#### ADR Calculation

For the ADR calculation module, 3 algorithms were implemented:

- Local energy deposition (LED). The energy emitted by charged particles is assumed to be fully deposited within the local voxel. A scaling factor is generated for the energy emitted by charged particles per megabecquerel. To compute the absorbed dose, this factor is multiplied by the activity within the voxel or VOI and divided by its corresponding mass, derived from the CT image.
- Convolution (CONV). This assumes a homogeneous medium. A fast Fourier transform algorithm ensures fast computing times. The activity image and the voxel *S* value (VSV) kernel must have identical voxel sizes. Precomputed VSV kernels in water and other media are available from the OpenDoseDVKData repository (https://gitlab.com/opendose/opendosedvkdata) for the supported radionuclides, covering voxel dimensions from 0.1 mm to 6.0 mm and a kernel size of 99 × 99 × 99 voxels. OD3D also allows importing external VSV kernels if they comply with the required formatting and naming conventions (see OD3D User’s Manual).
- Monte Carlo (MC). GATE version 9 (16) was chosen because of its widespread availability. The validation for voxel-based absorbed dose calculations in nuclear medicine therapy was presented elsewhere (17). As GATE inclusion in OD3D was considered impractical, the current project focused on generating a GATE input script in OD3D (based on activity/density maps) and on the automated integration of the GATE output into OD3D.

Nuclear decay data from the International Commission on Radiological Protection Publication 107 (18) were used to generate the scaling factor applied in the LED algorithm. For MC simulations, decay data are handled by the GATE ion source. For CONV, VSVs must be available (see OD3D User’s Manual).

Local density correction, applicable to LED and CONV, is possible based on the voxel density. For LED, this is an exact approach. Conversely, for CONV, it is an approximation because the energy spatial distribution is still computed using the density selected for the VSV. If density correction is not selected, ADRs are computed assuming a homogeneous water medium. A recent study reported the comparison of ADR calculation algorithms in OD3D (19).

#### Time-Dependent Curve Fit and Integration

The tools provided by the Python library *lmfit* were initially used for the fitting module and remain available as an option. The project’s collaborative nature allowed the integration of NUKFIT (20) features as an additional option for this module:

- *lmfit* supports monoexponential, biexponential, triexponential models.
- NUKFIT supports the list of functions provided by Hardiansyah et al. (21).

The initial step is to generate activity or ADR tables from the different VOIs and time points. Next, models with fewer fitting parameters *K* than the number of available time points *N* can be selected (*N* > *K*). This setting allows calculating the uncertainty of the fitted parameters (13). The interface also offers an option called Force Zero to restrict the selection to models that fulfil the condition *f*(*t* = 0) = 0.

Both options include trapezoidal and automated fit modes. In the trapezoidal mode, the tail effective half-life is calculated from the last 2 points. The automated fit mode involves selecting the best model through a structured process. All available models are fitted, and only those that meet the goodness-of-fit criteria (20, 22) are considered. These criteria ensure that the fitted parameters’ coefficient of variation (CV) remains below 50%. A warning is displayed if any of the absolute values of the off-diagonal elements in the correlation matrix exceed 0.8. For models that pass the goodness-of-fit criteria, the corrected Akaike information criterion is calculated, and model selection is based on the highest Akaike weight (20, 22). For this approach, the number of measurements must be larger than or equal to the number of parameters + 2 (absolute weighing in (23)).

If the corrected Akaike information criterion cannot be determined for all models meeting the goodness-of-fit criteria, the selection is based on the Bayesian information criterion, and the model with the lowest Bayesian information criterion value is selected. Automated fit is the default setting, but users can manually select a specific fitting function if desired.

### Validation

The MRTDosimetry project dataset (24) was used to validate some OD3D modules. This dataset provides a set of phantom scans, including Jaszczak, NEMA, and a 4-organ phantom (liver, spleen, kidneys, liver tumor) for which activity, TIA, and absorbed doses are known (ground truth).

#### Calibration

The calibration module was used to calculate the camera sensitivity factor and the RC curve. Input parameters were based on Tran-Gia et al. (12).

Sensitivity was determined using the extended source volume method (12). The Jaszczak phantom diameter (21.6 cm) was extracted from a profile in the axial view of the CT image, and the theoretic volume (6,900 mL) was used. RCs were obtained by applying a volume-based threshold option on the SPECT image.

The camera sensitivity factor and RC (for the 4-organ phantom) were compared with those obtained by Tran-Gia et al. for the same system and reconstruction protocol (Supplemental Fig. 2A; Figs. 4 and 5 in ref (12); supplemental materials are available at http://jnm.snmjournals.org).

#### Dosimetry

For dosimetry validation, the 4-organ phantom was used (25). It includes reconstructed SPECT/CT images at 6 time points (1, 4, 24, 40, 72, and 144 h).

In OD3D, no registration was needed because SPECT images were prealigned to the first CT image. Segmentation was performed on the first CT image and propagated. The ADR was computed using the CONV algorithm in a homogeneous water medium. Curve-fitting models were automatically selected for most VOIs. The healthy liver and tumor models were manually adjusted to ensure the curve to start at zero. The ACT and ADR workflows were used to estimate TIA and absorbed dose, respectively. RC from the Calibration module were used to correct for PVE. Results were compared against the ground truth.

### Clinical Dosimetry Example

An example of OD3D clinical application is presented using patient A (John Doe) from the 2021 Society of Nuclear Medicine and Molecular Imaging (SNMMI) Dosimetry Challenge (26). The test focused on reconstructed ^177^Lu SPECT/CT images acquired at 4 time points (4, 24, 48, and 120 h) after administration of 7,210 MBq of [^177^Lu]Lu-DOTATATE. As SPECT images were already quantified in becquerels per milliliter, no sensitivity calibration was required in OD3D. For the challenge, SPECT images were corrected for physical decay; however, for OD3D, this correction is not required, and thus, images were restored to the precorrection state. No PVE correction was implemented, as no phantom or RC curve was provided.

Task 1 of the challenge (dosimetry from multiple reconstructed SPECT/CT images) was addressed using the automated ADR workflow. Automated segmentation of the OARs (liver, kidneys, and spleen) was performed at each time point (without registration), as detailed in Table 1. Automated fit model selection was used and checked visually. After completion of the automated ADR workflow for OARs, 2 lesions were segmented on the SPECT image acquired at the first time point (4 h), using a 40% local maximum threshold. For the subsequent time points, a volume-based threshold was used to generate segmentations at the corresponding uptake locations. ADRs were calculated using LED (automated workflow) and then CONV algorithms with density correction.

Task 4 (dosimetry from multiple reconstructed SPECT/CT images + VOIs) was performed using the ADR workflow without registration, by importing the segmentations provided at each time point. Segmentations were extracted from the mask volumes supplied in the challenge dataset. The CONV algorithm was used for ADR calculations, and automated model selection was maintained.

Task 5 (VOIs + TIA image) was performed by importing into OD3D the TIA image and the segmentation provided for the first time point. Voxel values in the TIA image were converted to match the kernel units of the CONV algorithm (mGy·MBq^−1^·s^−1^). Then, the mean absorbed dose values for each VOI were extracted from the generated absorbed dose map.

To assess the segmentation impact on the absorbed dose results between Task 1 and Task 4, Dice scores were estimated for each segmentation pair at each time point, using the segmentation from Task 4 as reference.

Relative difference was computed to quantify the deviation of each OD3D result from a reference value (e.g., the median of the distribution). The CV was used to assess the variability within OD3D results. Additionally, a *z* score (27) was used to quantify the deviation of each OD3D result relative to the distribution of values reported by the challenge participants (28).

## RESULTS

### Validation

#### Calibration

For the phantom study, the camera sensitivity factor determined by the OD3D calibration module was 42.82 cps/MBq.

Equation 1 represents the RC curve derived from the calibration module using the NEMA phantom dataset. The maximum relative difference between the OD3D-derived RCs and those obtained using the RC curve provided previously (12), for the 5 volumes in the phantom dataset, was 2% (Table 2).

$$\text{RC}=1-\frac{1}{1+{\left(\frac{v}{2.53}\right)}^{0.41}}.$$ Eq. 1

**TABLE 2.** Segmented Volumes in OD3D for 4-Organ Phantom of MRTDosimetry Project*

VOI	Volume (cm^3^)	RC
Left kidney	137 (2)	0.84 (2)
Right kidney	116 (4)	0.83 (2)
Healthy liver	1,287 (−2)	0.93 (2)
Spleen	125 (0)	0.83 (2)
Tumor	16 (1)	0.68 (2)

* Relative differences from ground truth are shown in parentheses. RCs were calculated using Equation 1 obtained from calibration module in OD3D. Relative differences (% within brackets) were computed using the equation from Tran-Gia et al. (12).

#### Dosimetry

The dosimetry results for the 4-organ phantom are presented in Table 3. The TIA and absorbed dose calculated with OD3D for the 4-organ phantom were evaluated with and without RCs, using the ground truth as reference. Without RCs, the results showed a systematic underestimation in all organs, with deviations ranging from −7% to −20% for both TIA and absorbed dose. The RC application improved the accuracy for the 4 organs (deviations between −3% and 0%). In the tumor, the RCs led to an overestimation, with deviations of 30% for TIA and 32% for absorbed dose. The total processing time for the phantom dosimetry was approximately 30 min.

**TABLE 3.** TIA and Absorbed Dose Results Obtained with OD3D for 4-Organ Phantom of MRTDosimetry Project*

	Without RC		With RC	
VOI	TIA (MBq·h)	Absorbed dose (Gy)	TIA (MBq·h)	Absorbed dose (Gy)
Left kidney	4,626 (−18)	2.96 (−18)	5,526 (−2)	3.54 (−2)
Right kidney	3,839 (−18)	2.89 (−20)	4,639 (−1)	3.49 (−3)
Healthy liver	22,730 (−9)	1.58 (−7)	24,496 (−2)	1.70 (0)
Spleen	6,252 (−20)	4.36 (−19)	7,515 (−4)	5.24 (−3)
Tumor	4,675 (−11)	25.23 (−10)	6,870 (30)	37.07 (32)

* Results are presented with and without application of RCs. Relative differences from ground truth are shown in parentheses.

### Clinical Dosimetry Example

**FIGURE 2.**
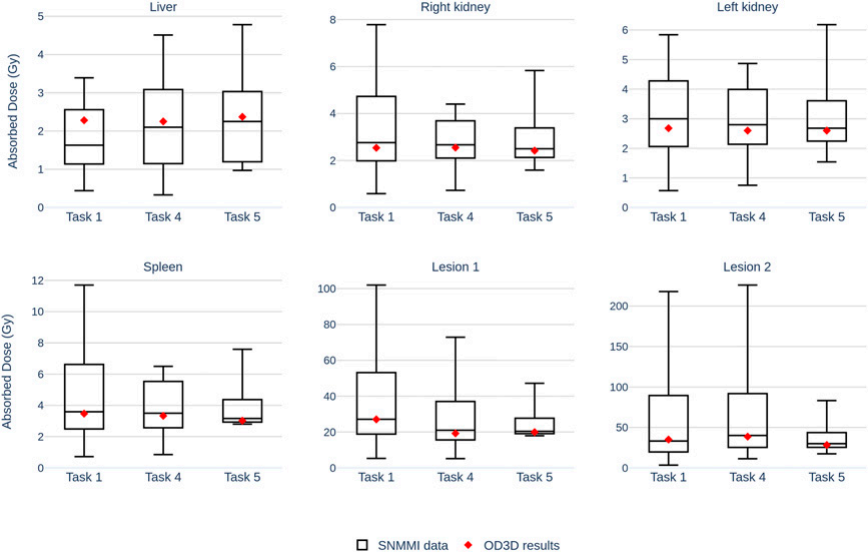
Absorbed dose results obtained with OD3D (red diamonds) and box plots showing results reported in supplemental data of SNMMI dosimetry challenge (28). Task 1 reflects result variability when participants perform dosimetry using different approaches for segmentation, registration, absorbed dose calculation, and time fitting/integration. In Task 4, VOI segmentations were provided. In Task 5, VOI segmentations and TIA were provided.

**FIGURE 3.**
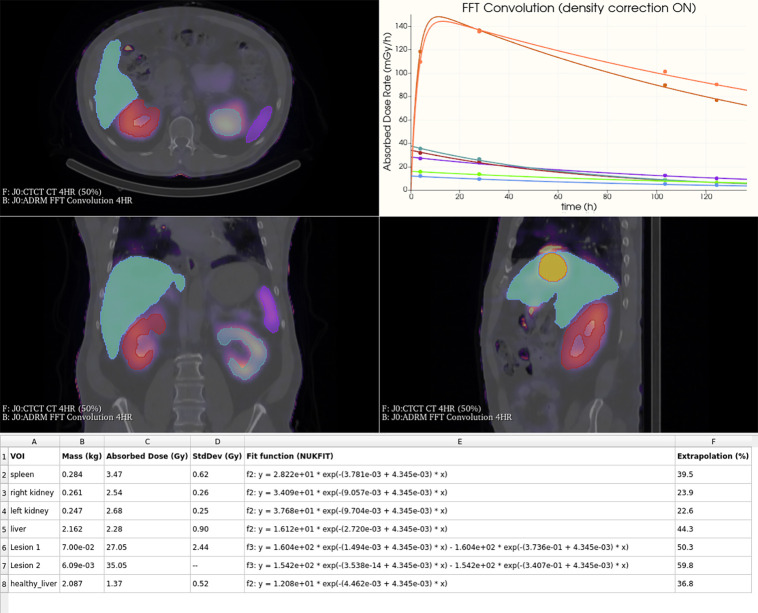
Results generated by OD3D. Plot displays mean ADR (mGy/h) values calculated using fast Fourier transform (FFT) convolution with density correction (as indicated in chart title) for each VOI at each time point. Best-fitting curves, obtained through NUKFIT model selection, are shown. Table (bottom) provides detailed information for each VOI: mass, absorbed dose obtained from area under curve of fitting function, SD (StdDev) of area under curve, fit function, and extrapolation percentage (i.e., ratio of extrapolated area to the total area under curve).

Figure 2 presents the absorbed dose results obtained with OD3D. SNMMI participants’ data were taken from the second part of the dosimetry challenge study (28). The OD3D results were consistently close to the median values and remained well within the interquartile ranges for all organs and lesions evaluated. Absolute *z* scores were systematically less than 1, indicating that no value differed by more than 1 SD from the mean value. The total processing time for Task 1 was approximately 10 min. The results displayed on the OD3D interface are shown in Figure 3.

For OARs, the highest deviation was observed in Task 1 for the liver, for which OD3D gave an absorbed dose of 2.28 Gy and the median SNMMI value was 1.63 Gy. The CV across tasks for OD3D was 4% for liver, 3% for right kidney, 1% for left kidney, and 7% for spleen.

For the 2 lesions, the CV across tasks was higher: 19% for lesion 1 and 16% for lesion 2. For lesion 1, the greatest deviation occurred between Task 1 and Task 4 (27 Gy to 19 Gy), whereas results were less variable between Task 4 and Task 5 (19 Gy to 20 Gy). Conversely, lesion 2 exhibited greater variability between Task 4 and Task 5 (39 Gy to 29 Gy), whereas results remained relatively stable between Task 1 and Task 4 (35 Gy to 39 Gy).

Table 4 shows the high variability in lesion segmentation between Task 1 and Task 4 for both lesions. For lesion 1, the segmented volume in Task 1 was up to 43% larger than that in Task 4, whereas for lesion 2, it was 62% smaller. In contrast, organ segmentations remained consistent across tasks, with variations ranging from 0.6% to 10%.

**TABLE 4.** Summary of Segmentation Agreement Metrics for All VOIs for Patient A in SNMMI Dosimetry Challenge Across 4 Imaging Time Points: 4, 24, 108, and 120 Hours*

VOI	Dice score
Spleen	0.94 (1.9)–0.96 (10.6)
Right kidney	0.93 (2.7)–0.94 (5.7)
Left kidney	0.94 (3.6)–0.95 (6.9)
Liver	0.97 (0.6)–0.97 (2.6)
Lesion 1	0.73 (42.4)–0.73 (42.9)
Lesion 2	0.63 (−62.4)–0.75 (−55.3)

* Minimum and maximum values of Dice score and relative volume difference (% within brackets) between our segmentation (Task 1) and reference segmentation (Task 4) are reported.

Additional CDW combinations, such as rigid and elastic registration, and absorbed dose calculations using the LED and MC algorithms are presented in the supplemental materials. CDW variability was generally low for organs (maximum CV of 10% for the spleen) and lesion 1 (CV = 2%), whereas lesion 2 (the smaller one) showed higher variability (CV = 30%). When the impact of registration was assessed, the best agreement with the median (−2% relative difference for lesion 2) was obtained when no registration was applied, and segmentation was performed independently at each time point; in contrast, the ACT workflow with elastic registration showed the largest deviation (−58% for lesion 2). Absorbed dose results were in agreement with the median of the participant results (29), with a maximum absolute relative difference of 7% for lesion 2 using the LED algorithm (excluding the liver, as the healthy and whole liver segmentations are merged in the results). Values were consistent across algorithms, with LED slightly lower or in line with CONV and MC.

## DISCUSSION

OD3D was validated using image datasets from the MRTDosimetry project. The sensitivity calibration and recovery curve produced by OD3D showed excellent agreement (within 2%) with the reference data reported by Tran-Gia et al. (12). For the organ compartments in the 4-organ phantom, the calculated TIAs and absorbed doses were also in excellent agreement with the ground truth, with relative differences below 3%.

For the tumor compartment, the OD3D curve-fitting model tended to overestimate the tumor TIA compared with the ground truth activity data, due to a higher effective half-life estimated for the washout phase (Supplemental Fig. 1). This overestimation may be attributed to by the complex pharmacokinetic model used as ground truth (30), which might be the reason for the observed 32% overestimation in the tumor absorbed dose.

Our results are within the range of those produced by the SNMMI dosimetry challenge participants and were in excellent agreement with the median values for each task. The observed discrepancy for liver in Task 1 (Fig. 2) may be explained by the fact that participants performed their own segmentation in Task 1 and might have focused on the healthy liver, whereas the provided segmentations used in later tasks considered the whole liver. OD3D results for healthy liver (Supplemental Fig. 2) are consistent with these observations. Overall, the results for OARs were highly consistent across all 3 tasks.

For lesions, higher variability was observed. The variations seen from Task 1 to Task 4 can be attributed mainly to differences in segmentation approaches. Dice scores (Table 4) confirmed the greater variability of lesion segmentations. For lesion 2, the variability observed between Task 4 and Task 5 can be explained by differences in the pharmacokinetic model fitting. The TIA map provided in Task 5 was generated by an external software using voxel-level curve fitting, whereas OD3D used an organ-level fitting model in Task 4.

The ADR calculation algorithms in OD3D have been extensively tested (19). ADRs are calculated at the voxel level at each time point, but the absorbed dose is averaged over the VOI because uncertainties associated with voxel-based registration over time points cannot be assessed.

OD3D is a collaborative project that associates a growing number of partners. Not being confined to a single laboratory allows benefiting from the input of groups with expertise in a broader range of domains relevant to clinical dosimetry. This is an asset to increase software sustainability. OD3D is available as an official 3D Slicer module, thereby facilitating its installation on various computing platforms.

OD3D User’s Manual is available on the GitLab platform, and a YouTube channel (https://www.youtube.com/@OpenDose3D) guides users in their first steps.

This project, distributed under the Apache License 2.0, allows testing approaches or algorithms that are not always supported in existing software tools. As the source code is publicly available, features tested in OD3D may be freely integrated in existing software (academic or commercial) tools. The development road map is discussed among the collaboration members and is presented at the end of OD3D User’s Manual.

Although OD3D offers a flexible and open framework for fully patient-specific dosimetry, some limitations remain. Currently, only the LED approach is available in the activity workflow. The software requires reconstructed images as input, supporting only 3D dosimetry; hybrid (planar/SPECT) dosimetry is not implemented. Single-time-point dosimetry is only available by manually entering an effective decay time (e.g., derived from population-based data). Future developments aim to introduce more robust approaches for single-time-point dosimetry and enable the integration of radiobiologic parameters in the dosimetry workflows.

OD3D allows comparing different CDW implementations in different software tools. This is crucial because of the increasing number of publications on the impact of dosimetry in therapeutic nuclear medicine (31). These studies are based on different CDWs, limiting the possibility of pooling dosimetry results. As OD3D allows storing intermediate results and implementing different CDWs, it can help to compare and pool results obtained using different dosimetric methodologies.

## CONCLUSION

OD3D enables image-based, patient-specific dosimetry for software testing, research purposes, and academic clinical trials. The software was validated against phantom data and tested using patient data from the SNMMI dosimetry challenge.

OD3D represents a major achievement of the OpenDose collaboration. Its open-source and collaborative nature is designed to support the project’s long-term sustainability and promote continuous development.

## DISCLOSURE

The OD3D project was mostly developed during Vergara-Gil’s (http://thesesups.ups-tlse.fr/5458/) and Fragoso-Negrín’s doctoral project. José-Alejandro Fragoso-Negrín is sponsored by DOSIsoft within the PhD contract CIFRE no. 201273A10. Manuel Bardiès and Lore Santoro supervise José-Alejandro Fragoso-Negrín, sponsored by DOSIsoft within the PhD contract CIFRE no. 201273A10. Manuel Bardiès is acting as a consultant advisor for Clario. Gerhard Glatting is acting as a consultant advisor for ITM and ESQlabs. Deni Hardiansyah is acting as a consultant advisor for ITM. Emmanuel Deshayes received fees from Janssen, Novartis, and GE HealthCare. Deni Hardiansyah is supported by the PUTI Q1 grant 2025 (PKS-200/UN2.RST/HKP.05.00/2025) from Universitas Indonesia. This work was partly performed within the framework of the PhD CIFRE contract no. 201273A10. The IRCM team (José-Alejandro Fragoso-Negrín, Lore Santoro, Susana Veloza-Awad, Emmanuel Deshayes, and Manuel Bardiès) is an “Équipe Labellisée Ligue Contre le Cancer”. No other potential conflict of interest relevant to this article was reported.

## Notes

KEY POINTS **QUESTION:** Can a free, collaborative open-source tool perform image-based dosimetry using clinical datasets? **PERTINENT FINDINGS:** OD3D implements several CDWs. It is a reliable tool for benchmarking dosimetry software, conducting clinical dosimetry trials, and comparing dosimetry approaches. **IMPLICATIONS FOR PATIENT CARE:** With OD3D, dosimetric results obtained with different approaches and software can be compared and thus optimized and improved, enabling individualized dosimetry, which may benefit patients by predicting associated risks.
